# Lissajous Scanning Two-photon Endomicroscope for *In vivo* Tissue Imaging

**DOI:** 10.1038/s41598-019-38762-w

**Published:** 2019-03-05

**Authors:** Daniel Youngsuk Kim, Kyungmin Hwang, Jinhyo Ahn, Yeong-Hyeon Seo, Jae-Beom Kim, Soyoung Lee, Jin-Hui Yoon, Eunji Kong, Yong Jeong, Sangyong Jon, Pilhan Kim, Ki-Hun Jeong

**Affiliations:** 1 Department of Bio and Brain Engineering, KAIST, Daejeon, 34141 Republic of Korea; 2 Biomedical Science and Engineering Interdisciplinary Program, KAIST, Daejeon, 34141 Republic of Korea; 3 Department of Biological Sciences, KAIST, Daejeon, 34141 Republic of Korea; 4 KAIST Institute of Health science and technology, Daejeon, 34141 Republic of Korea; 5 KAIST Institute for the BioCentury, Daejeon, 34141 Republic of Korea

## Abstract

An endomicroscope opens new frontiers of non-invasive biopsy for *in vivo* imaging applications. Here we report two-photon laser scanning endomicroscope for *in vivo* cellular and tissue imaging using a Lissajous fiber scanner. The fiber scanner consists of a piezoelectric (PZT) tube, a single double-clad fiber (DCF) with high fluorescence collection, and a micro-tethered-silicon-oscillator (MTSO) for the separation of biaxial resonant scanning frequencies. The endomicroscopic imaging exhibits 5 frames/s with 99% in scanning density by using the selection rule of scanning frequencies. The endomicroscopic scanner was compactly packaged within a stainless tube of 2.6 mm in diameter with a high NA gradient-index (GRIN) lens, which can be easily inserted into the working channel of a conventional laparoscope. The lateral and axial resolutions of the endomicroscope are 0.70 µm and 7.6 μm, respectively. Two-photon fluorescence images of a stained kidney section and miscellaneous *ex vivo* and *in vivo* organs from wild type and green fluorescent protein transgenic (GFP-TG) mice were successfully obtained by using the endomicroscope. The endomicroscope also obtained label free images including autofluorescence and second-harmonic generation of an ear tissue of Thy1-GCaMP6 (GP5.17) mouse. The Lissajous scanning two-photon endomicroscope can provide a compact handheld platform for *in vivo* tissue imaging or optical biopsy applications.

## Introduction

Advanced optical microscopy such as optical coherence tomography (OCT)^1,2^, confocal microscopy (CM)^3–7^, or two-photon microscopy (TPM)^8–11^ that enables bioimaging with intracellular or cellular level resolution has been actively miniaturized for *in vivo* imaging applications such as optical biopsy^1–7,9,12–14^ or *in vivo* brain imaging of freely behaving animals^8,15,16^. Unlike OCT with non-specificity and relatively low resolution^17^ or CM with low penetration and relatively high photobleaching^18^, TPM has several advantages such as deeper penetration depth, inherent optical sectioning capabilities, and low photobleaching and photodamage in a small volume of the focal plane^18–20^.

Endomicroscopic TPM has been recently demonstrated by using micromirrors^15,21–24^ and fiber scannners^8,9,25–32^. However, the endomicroscopic TPM are still challenging due to compact packaging, stability of scanners, high frame rate, or group delay dispersion. Micromirror based scanning probes requires additional optical components to achieve forward-viewing endomicroscopy, which precludes from reducing the diameter of the whole probes into smaller than 5 mm in a diameter^21,33^. A fiber-optic scanning probe can be reduced smaller than 3 mm, which facilitates the insertion into the working channel in an endoscope^9,31^. A fiber scanner consists of a piezoelectric (PZT) actuator and a single fiber, generating three different scanning patterns; raster^27,28^, spiral^9,25,29–31^, and Lissajous^8,26,32^. The resonant-based spiral scanners are successful regarding with small form factor and fast image acquisition^25^, but the fiber scanner with spiral pattern is usually venerable due to eccentricity of PZT tube, which causes a mechanical coupling phenomenon coupling between the x-axis and the y-axis^34^. However, Lissajous scanners distinctly emphasize high illumination uniformity and low photodamage in the center region^32^. Besides, the Lissajous scanning solves the mechanical coupling problem because it drives the x-axis and y-axis at different frequencies. Recent development of the micromachined tethered silicon oscillator (MTSO) attached on a fiber tip can readily generate Lissajous scanning for confocal endomicroscopy^35–38^. However, the Lissajous scanner based on the PZT has not been yet applied for two-photon endomicroscopy. In addition, the other conventional Lissajous two-photon endomicroscopies^8,26,32^ have been demonstrated at speeds below 3 frames.

**Figure 1:**
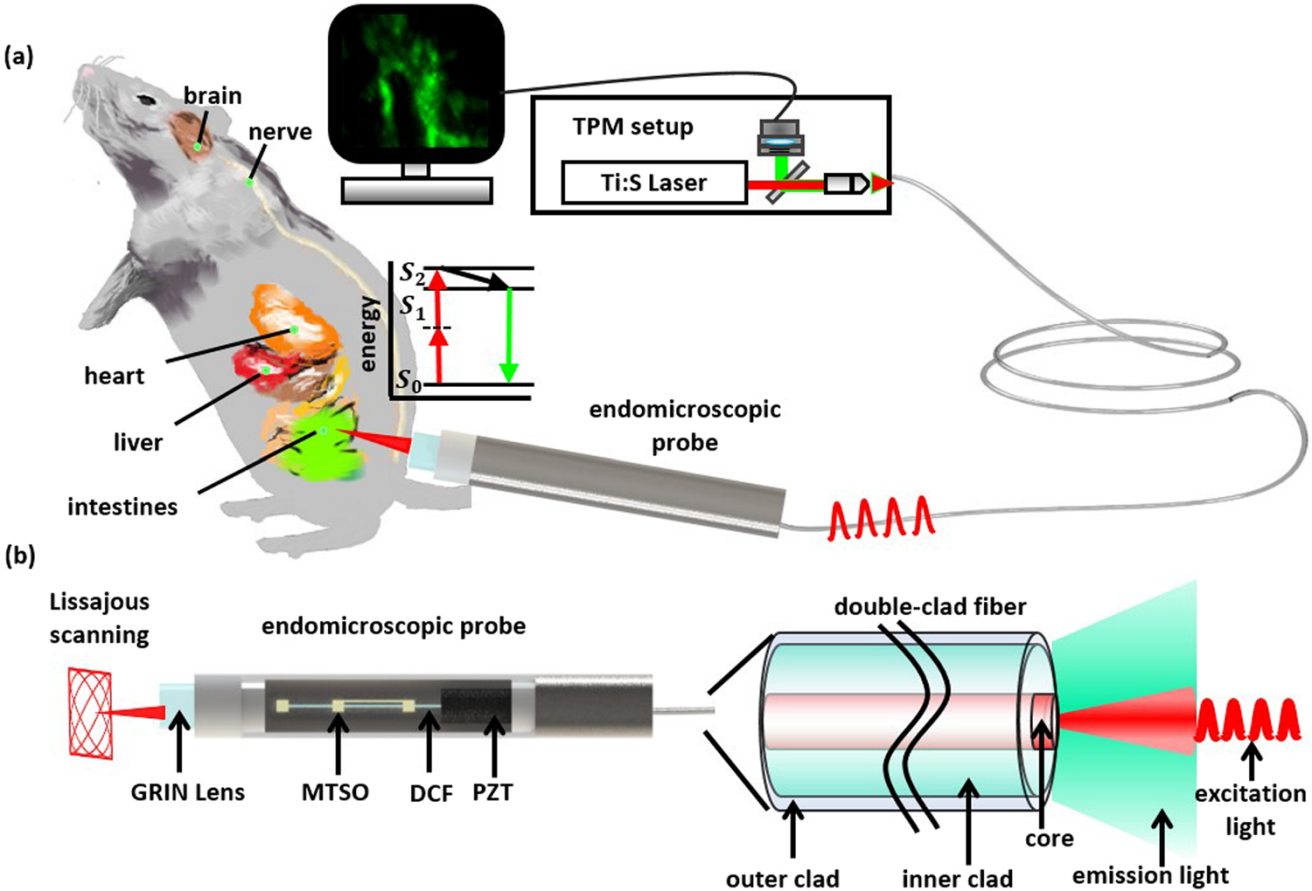
Conceptual design of a Lissajous two-photon endomicroscope. (**a**) A schematic illustration for a Lissajous two-photon endomicroscope for real-time and *in vivo* tissue imaging for small animals. (**b**) Endomicroscopic probe configuration containing a GRIN lens and a quadrupole piezoelectric (PZT) fiber scanner with a micromachined tethered silicon oscillator (MTSO), coupled with a double-clad fiber (DCF) that delivers femtosecond pulse excitation light through the single-mode core and collects two photon fluorescent emission light through the multimode inner clad.

Here we report a fully packaged two-photon endomicroscope for *in vivo* cellular and tissue imaging using Lissajous fiber scanner. Figure 1a shows the schematic diagram of Lissajous two-photon endomicroscope and its application on *in vivo* tissue imaging. The TPM setup couples femtosecond pulse laser into the Lissajous fiber probe and visualizes tissue images. Figure 1b also shows the schematic design of endomicroscopic probe comprising a GRIN lens and a quadrupole piezoelectric (PZT) fiber scanner based on a MTSO coupled a double-clad fiber (DCF), which delivers femtosecond pulsed laser and increases the collection of fluorescence emission signals through both the core and the inner clad. The fiber scanner obtained real-time *ex vivo* and *in vivo* images at 5 frame rate using the frequency selection rule for high definition and high frame rate (HDHF)^36^ Lissajous scanning.

## Results and Discussions

### Resonant Lissajous fiber scanner design and optomechanical properties

**Figure 2:**
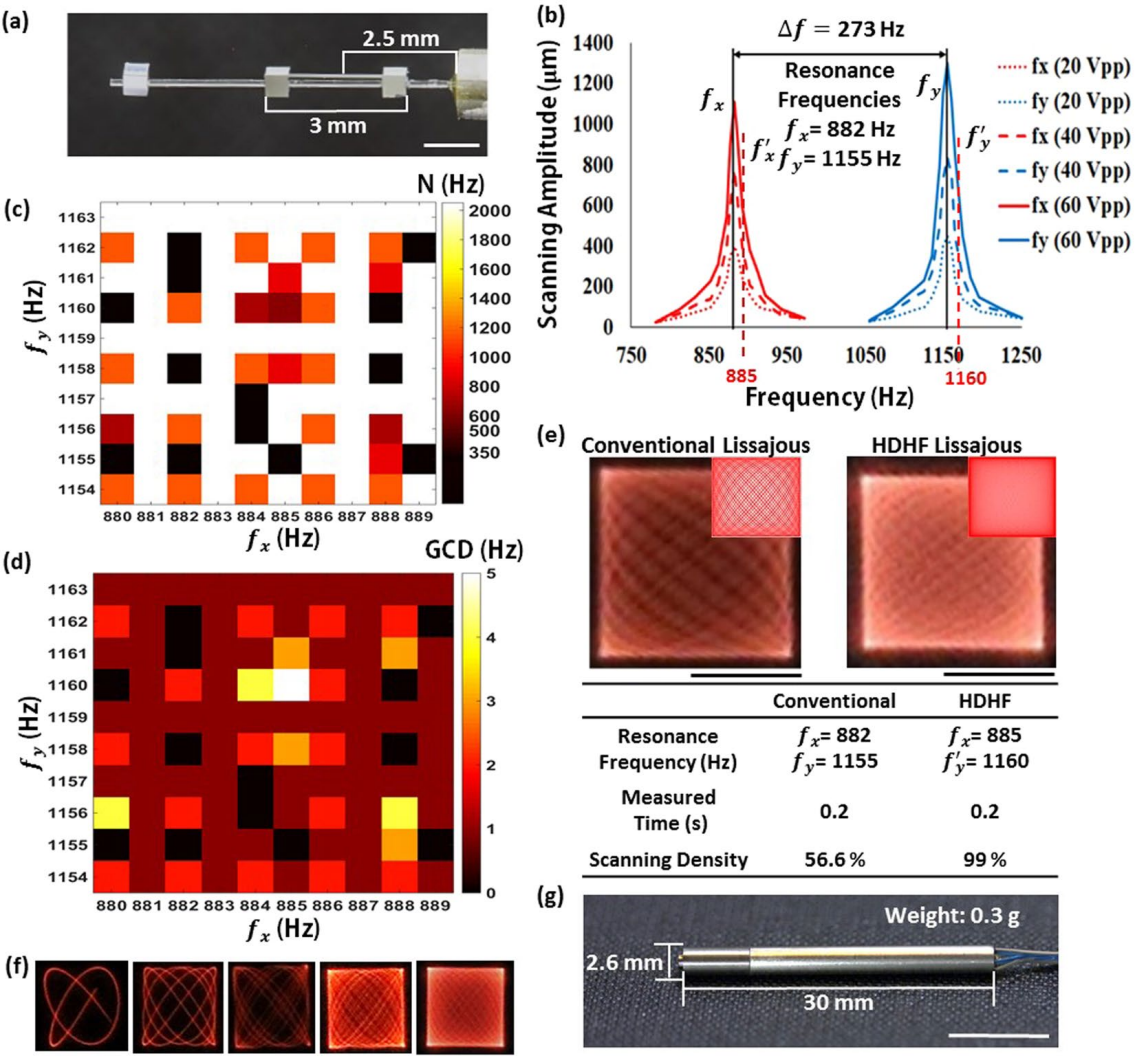
Resonant Lissajous fiber scanner design and optomechanical properties. (**a**) Optical image of the Lissajous fiber scanner composed of a PZT tube, a DCF, and a MTSO. The length of the MTSO was 3 mm, which center was 2.5 mm away from the PZT tube. (Scale bar = 1 mm). (**b**) Frequency response and corresponding scanning amplitudes of the fiber scanner. The resonant frequencies of x-axis and y-axis were 882 Hz and 1,155 Hz (black lines), respectively. The scanning amplitudes of x-axis and y-axis were 400 μm and 450 μm at 20 V_pp_, respectively. 885 Hz and 1,160 Hz were selected for x-axis and y-axis resonance frequencies (red dotted lines) to apply high definition and high frame-rate (HDHF) Lissajous scanning. (**c**) A color-map of total lobe number (N) depending on scanning frequencies, where the maximum (N $\ge$ 2,000) and the minimum (N $\le$ 350) indicate in yellow to black color, respectively. (**d**) A color map of the great common division (GCD) depending on the scanning frequencies. The specific areas, where N is lower than 350, are filled in black color. (**e**) Difference between conventional and HDHF Lissajous scanning. Scanning density at the 0.2 s measured time was increased from 56.6% to 99% by applying the frequency selection rule. (Scale bar = 200 μm). The optical images of Lissajous scanning pattern are shown with simulated scanning trajectories. (**f**) Time-lapse images for stable Lissajous scanning of 885 Hz and 1160 Hz were captured for 2.5 msec, 10 msec, 20 mesc, 50 msec, and 100 msec. The fill factors are 3.4%, 12.5%, 24.6%, 53.4%, and 81% for each time in order. (**g**) Optical image of the compactly packaged probe and its dimension: 2.6 mm in diameter, 30 mm in length, and 0.3 g in weight. (Scale bar = 5 mm).

The MTSO attached on the DCF, which has 7 mm long, differentiates the bi-directional resonant frequencies to generate Lissajous scanning. The MTSO consists of a silicon mass and a microspring which has two silicon masses with a 3 mm long structure. The silicon mass was attached on the distal end of the optical fiber to increase effective mass, and the center of microspring was set to 2.5 mm away from the PZT tube (Fig. 2a). Figure 2b shows the frequency response and the scanning amplitudes of Lissajous fiber scanner depending on various driving voltages. The resonant frequencies of x-axis (f_x_) and y-axis (f_y_) were 882 Hz and 1,155 Hz, respectably. The MTSO separated resonant frequencies 273 Hz and eliminated the mechanical coupling phenomenon. The scanning amplitudes of x-axis and y-axis were 400 μm and 450 μm at 20 V_pp_, respectively and the scanning amplitude ratio between x-axis and y-axis was 0.88 at 20 V_pp_. A particular set of scanning frequencies using the selection rule can increase a frame rate and scanning density that allows the HDHF Lissajous scanning [24]. Figure 2c shows a color-map of total lobe number (N) depending on the scanning frequencies, where the maximum (N $\ge$ 2,000) and the minimum (N $\le$ 350) indicate in yellow and black color, respectively. Figure 2d describes a color map of the great common division (GCD) depending on the scanning frequencies, and the regions, where N is lower than 350, are filled in black color. The scanning frequencies for HDHF Lissajous scanning were selected as 885 Hz and 1,160 Hz for x-axis and y-axis, respectively for N = 409 and GCD = 5. The comparison between the conventional and the HDHF scanning methods is shown in Fig. 2e. The scanning frequencies of conventional Lissajous scanning were selected as the exact resonance frequencies (f_x_ = 882 Hz and f_y_ = 1,155 Hz). In contrast, HDHF Lissajous scanning can be operated at pseudo resonance frequencies (f_x_′ = 885 Hz and f_y_′ = 1,160 Hz) near the resonance. The scanning density of the HDHF Lissajous scanning (99%) was increased compared to that of conventional Lissajous scanning (56.6%) at the same measured time (0.2 s). Time-lapse images for stable Lissajous scanning of 885 Hz and 1160 Hz were captured for 2.5 msec, 10 msec, 20 mesc, 50 msec, and 100 msec as shown in Fig. 2f. The fill factors are 3.4%, 12.5%, 24.6%, 53.4%, and 81% for each time in order. The scanning speed of Lissajous scanning was in the range of approximately 0.2 pixel/μs to 1 pixel/μs. The scan speed is relatively slow at the edge of the Lissajous pattern than other areas, but only about 2% of the total pixels have the scanning speed of 0.33 pixel/μs or less (see Supplementary Fig. S1). The Lissajous fiber scanner was fully packaged with a stainless housing tube and a lens tube including the achromatic miniature GRIN lens (MO-080-018-AC450-900, GRINTECH), which has magnification of 4.76 with image NA of 0.175 and objective NA of 0.8, and the optical image is shown in Fig. 2g. Fully packaged scanning probe had 2.6 mm in diameter, 30 mm in length, and 0.3 g in weight. The probe shows relatively small filed-of-view compared to the spiral pattern endomicroscope due to the size of the silicon mass and the objective mount, but the silicon mass produced a Lissajous pattern that improved frame rate and stability over the spiral.

### Lissajous two-photon endomicroscope system setup and optical resolution

**Figure 3:**
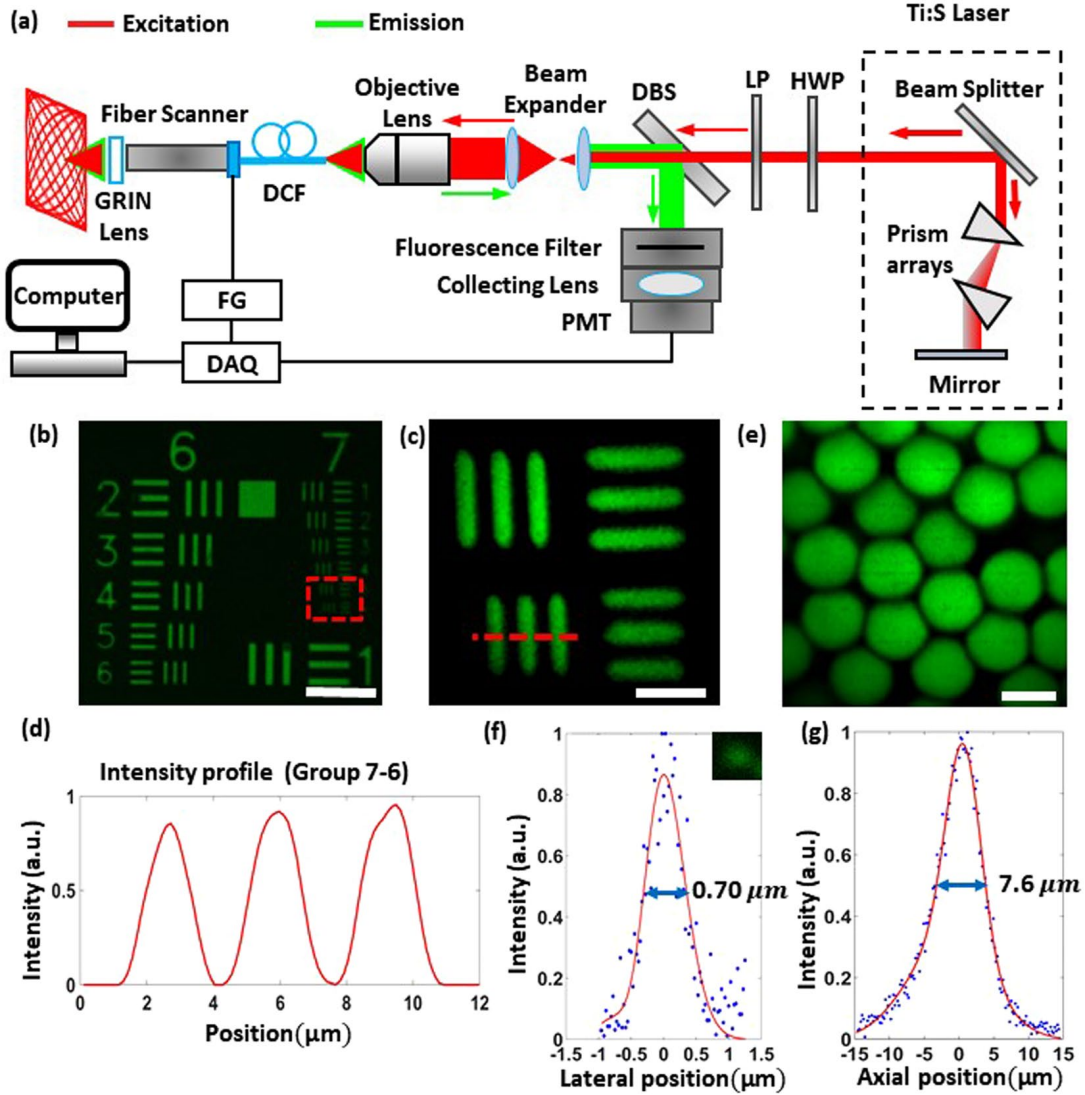
Lissajous two-photon endomicroscopic system setup and optical resolution. (**a**) System setup for the two-photon microscopic system and the Lissajous fiber scanner. HWP: half-wave plate, LP: linear polarizer, DBS: dichroic beam splitter, DAQ: signal digitizer and data acquisition, FG: function generator. (**b**) Microscope (Nikon, L-IM) fluorescence image of United States Air Force (USAF) test target patterned PDMS after coating fluorescein isothiocyanate (FITC). (Scale bar = 50 μm). (**c**) Two-photon fluorescence (TPF) image of the USAF pattern using the two-photon endomicroscope (group 7, element 5–6). (Scale bar = 10 μm). (**d**) TPF intensity line profile across the vertical bars of group 7, element 6. (Scale bar = 10 μm). (**e**) TPF image of 10 μm diameter fluorescent beads (G1000, ThermoFisher). (Scale bar = 10 μm). (**f**) TPF intensity profiles of 200 nm beads (QGC-200-1, Ocean NanoTech) show full-width at half-maximum (FWHM) of (f) 0.7 μm in lateral and (**g**) 7.6 μm in axial directions. The solid curves were Gaussian fitted to the data points. The excitation wavelength was 900 nm.

The endomicroscopic system setup is shown in Fig. 3a. A Ti:sapphire (Ti:S) laser (Chameleon vision II, Coherent) provides ultrashort laser pulses with 100 fs pulse width. The excitation laser was coupled into a DCF (SMM 900, Fibercore) using an objective lens (RMS X10, Thorlabs). To compensate the chromatic dispersion of the optical fiber, the laser was pre-chirped by a prism array inside the Ti:S laser. Because the maximum negative group delay dispersion of the prism arrays was 9,500 fs^2^, the length of the DCF was set to 17 cm (see Supplementary, Fig. S2). The optical resolution of the endomicroscope was characterized by using the customized United States Air Force (USAF) test target patterned PDMS fluorescein isothiocyanate (FITC) coating (see Supplementary, Fig. S3) and the 10 µm (G1000, Thermo Fishers Scientific) and 200 nm (QGC-200-1, Ocean NanoTech) beads, as shown in Fig. 3b–g. Figure 3b shows the microscope (L-IM, Nikon) fluorescence image of the customized USAF test target patterned PDMS (group 6 and 7). Two-photon fluorescence (TPF) image of the group 7, elements 5 and 6, which have line widths of 2.46 μm and 2.19 μm, respectively, was discernible using the endomicroscope (Fig. 3c). Figure 3d shows the intensity profile across the vertical bars of group 7, element 6. Figure 3e shows TPF image of 10 μm beads. The lateral and axial full-width at half-maximum (FWHM) of the intensity profiles of 200 nm fluorescence beads were 0.70 μm and 7.6 μm, respectively (Fig. 3f,g).

### Two-photon mouse tissue images

**Figure 4:**
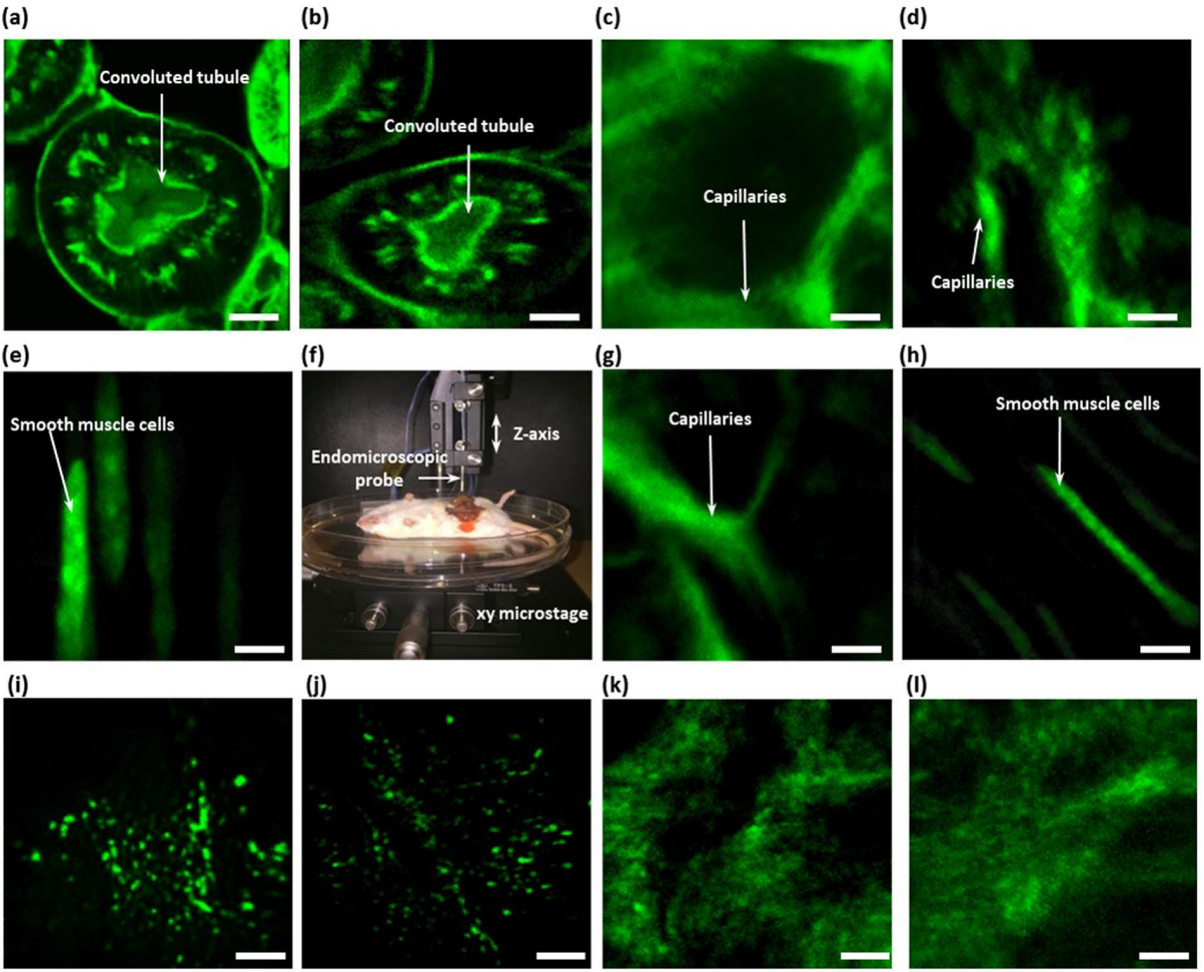
Two-photon mouse tissue images. (**a**,**b**) TPF images from a mouse kidney section stained with Alexa Fluor 488 wheat germ agglutinin (F-24630, Invitrogen). (**a**) TPF image of the commercial TPM (LSM 510, Zeiss). (**b**) TPF image obtained by the endomicroscope. (**c**–**e**) *ex vivo* images collected by endomicroscope. (**c**) Microvascular *ex vivo* image of a mouse small intestine after intravenous injection of FITC. (**d**) Microvascular *ex vivo* image of a mouse ear after intravenous injection of FITC. (**e**) *Ex vivo* green fluorescent protein (GFP) signal of smooth muscle cells in the small intestine. (**f**) *In vivo* mouse imaging setup. (**g**,**h**) *In vivo* images obtained by the endomicroscope. (**g**) Microvascular *in vivo* images of a mouse ear after injecting FITC. (**h**) *In vivo* GFP signal of smooth muscle cells in the small intestine. The excitation wavelength was 900 nm. (**I**,**j**) *Ex vivo* autofluorescence images from mouse ear. The excitation wavelength was 840 nm. (**k**,**l**) *Ex vivo* second-harmonic generation (SHG) images from collagens of mouse ear. The excitation wavelength was 880 nm. For the endomicroscope, all images were acquired at 5 frames/s with averaged five frames. (Scale bar = 10 μm).

Two-photon mouse tissue images were obtained from a stained mouse kidney section, miscellaneous *ex vivo* and *in vivo* organs, and label free images of *ex-vivo* mouse ear, as shown in Fig. 4. A mouse kidney section stained with Alexa Fluor 488 wheat germ agglutinin (F-24630, Invitrogen) was used to compare two-photon tissue images of a commercial TPM (LSM 510, Zeiss) and the endomicroscope (Fig. 4a,b). Both microscopic images, which show the convoluted tubules in kidney cells, were comparable, although the image obtained by the endomicroscope had five times faster frame rate and three times lower frame averaging. Wild type (BALB/c) mice and H2B green fluorescent protein transgenic (GFP-TG) mice were prepared to obtain *ex vivo* (Fig. 4c–e) and *in vivo* tissue imaging (Fig. 4g,h). In case of the wild type mouse, 1% FITC was injected into the mouse by intravenous injection to obtain microvascular images of each organ. In case of the H2B GFP-TG mouse, the green fluorescent protein (GFP) signals were obtained from nuclei in small intestine. Figure 4c and d show microvascular *ex vivo* images of the wild type mouse small intestine and ear, respectively. Figure 4e shows *ex vivo* GFP signals of smooth muscle cells in the GFP-TG mouse small intestine. After both mice were anesthetized, the mice were placed on the XYZ translation stage for *in vivo* tissue imaging (Fig. 4f). Figure 4g and h show microvascular *in vivo* image of the wild type mouse ear and in *vivo* GFP signals of smooth muscle cells in the GFP-TG mouse small intestine, respectively. Thy1-GCaMP6 (GP5.17) mouse was prepared to obtain label-free imaging (Fig. 4i–l). Figure 4I,j show *ex-vivo* autofluorescence images of mitochondrial NADH from the mouse ear and Fig. 4k,l show *ex-vivo* second-harmonic generation (SHG) images from collagens of the mouse ear.

We have demonstrated the Lissajous two-photon endomicroscope for *in vivo* tissue imaging. The attachment of MTSO on the DCF enables Lissajous scanning, which increases illumination uniformity and reduces photodamage, and applying the frequency selection rule leads to achieve up to 5 frames/s with 99% in scanning density. The image of the kidney convoluted tubule acquired from the endomicroscope was comparable to those from conventional TPMs, although the image from the endomicroscope had five times faster frame rate and three times lower frame averaging. Furthermore, the microvascular FITC and the GFP signals of *in vivo* images and label-free images demonstrate that the Lissajous two-photon endomicroscope can be utilized for *in vivo* cellular and tissue imaging.

## Methods

### Lissajous fiber scanner fabrication

The Lissajous fiber scanner consists of a PZT tube, a DCF, and a MTSO. The MTSO has a silicon mass of 0.5 mm × 0.5 mm × 0.4 mm and a microspring, which has two silicon masses with a 3 mm long and 50 µm width support structure. The MTSO were micro-machined by using 6-inch SOI wafer with a 40 μm thick top silicon layer, a 2 μm thick buried oxide layer, and a 400 μm thick bottom silicon layer. First, the bottom side of the SOI wafer was passivated with a thin aluminum layer to improve heat conduction and prevent leakages of helium cooling gas during a deep reactive ion etching (DRIE) process. Second, the top side of the wafer was patterned with photoresist and etched by using DRIE. Third, the top side of the wafer was passivated with parylene to prevent thermal stress during the next DRIE process. Fourth, the bottom side of the wafer was patterned with photoresist and etched by using DRIE. Fifth, the remained photoresist and passivation layers were completely removed by using plasma ashing and aluminum wet etching. The MTSO were released by removing the buried oxide layer using vapored HF and disconnecting silicon tethers with Joule heating^35^.

### Microscope setup

A Ti:sapphire laser (Chameleon vision II, Coherent) provided ultrashort laser pulses at 900 nm (initial pulse width ~ 100 fs) pre-chirped by an embedded prism array. The beam intensity was controlled by using an achromatic half-wave plate (AHWP05M-980, Thorlabs) and a calcite polarizer (10GL08AR.16, Newport). Dichroic beam splitter (FF705-Di01-25 × 36, Semrock) passed the laser pulses (900 nm/840 nm/880 nm) and reflected signal (509~525 nm for two-photon singal 420~440 nm for label free signal). To couple the laser to the core of DCF, the beam was initially expanded using two achromatic lenses (f = 160 mm, 67-334-INK, Edmund Optics), (f = 30 mm, 49–352-INK, Edmund Optics). The expanded beam was focused by using a microscope objective lens (RMS X10, Thorlabs) and coupled to the core of DCF (see Supplementary Fig. S4). The emission light from a sample passed a band pass filter (FF01-525/45–25, Semrock for two-photon signal and FF01-417/60–25, Semrock for label-free signal) to transmit only signal, and the signal was focused to the photomultiplier (R3788, Hamamatsu) using an achromatic lens (47–633-INK, Edmund Optics).

### Mouse preparation

Wild type (BALB/c), H2B GFP-TG, and GP5.17 mice were used to obtain *ex vivo*, *in vivo*, *and label-free* tissue imaging. For the *in vivo* imaging, both mice were anesthetized by using intraperitoneal injection of a mixture of zoletil (30 mg/kg) and xylazine (10 mg/kg). The mouse skin was shaved by hair clippers and removal cream before the surgical procedure for imaging. After a small incision on the abdominal skin of the anesthetized mice, small intestine was pulled out to minimize motion artifacts and imaged by using the endomicroscope. In case of wild type mice, 1% FITC-dextran (BCBF2730V, Sigma) was intravenously injected into the mice to visualize microvascular images. In case of the GP5.17 mouse, ears were cut out and imaged after shaving. This study was carried out in accordance with the ARRIVE (Animal Research: Reporting *In Vivo* Experiments) guidelines. All animal experiments were performed in accordance with the protocol approved by the Animal Care Committee of KAIST (protocol no. KA2018-06). All surgical procedures were performed under anesthesia, and all efforts were made to minimize the suffering.

## Supplementary information

Supplementary information

## References

[1] Liang KC (2018). Cycloid scanning for wide field optical coherence tomography endomicroscopy and angiography *in vivo*. Optica.

[2] Adler DC (2007). Three-dimensional endomicroscopy using optical coherence tomography. Nat Photonics.

[3] Polglase AL (2005). A fluorescence confocal endomicroscope for *in vivo* microscopy of the upper- and the lower-GI tract. Gastrointest Endosc.

[4] Shin HJ (2007). Fiber-optic confocal microscope using a MEMS scanner and miniature objective lens. Opt Express.

[5] Kiesslich R, Goetz M, Vieth M, Galle PR, Neurath MF (2007). Technology insight: confocal laser endoscopy for *in vivo* diagnosis of colorectal cancer. Nat Clin Pract Oncol.

[6] Sharma P (2011). Real-time increased detection of neoplastic tissue in Barrett’s esophagus with probe-based confocal laser endomicroscopy: final results of an international multicenter, prospective, randomized, controlled trial. Gastrointest Endosc.

[7] Hurlstone DP (2008). *In vivo* real-time confocal laser scanning endomicroscopic colonoscopy for the detection and characterization of colorectal neoplasia. Brit J Surg.

[8] Helmchen F, Fee MS, Tank DW, Denk W (2001). A miniature head-mounted two-photon microscope. high-resolution brain imaging in freely moving animals. Neuron.

[9] Myaing MT, MacDonald DJ, Li X (2006). Fiber-optic scanning two-photon fluorescence endoscope. Opt Lett.

[10] Murari K (2011). Compensation-free, all-fiber-optic, two-photon endomicroscopy at 1.55 mu m. Optics Letters.

[11] Liang, W. X., Hall, G., Messerschmidt, B., Li, M. J. & Li, X. D. Nonlinear optical endomicroscopy for label-free functional histology *in vivo*. *Light-Sci Appl* **6**, 10.1038/lsa.2017.82 (2017). 10.1038/lsa.2017.82 PMC5972527 29854567

[12] Seo YH, Hwang K, Jeong KH (2018). 1.65 mm diameter forward-viewing confocal endomicroscopic catheter using a flip-chip bonded electrothermal MEMS fiber scanner. Opt Express.

[13] Seo YH, Hwang K, Park HC, Jeong KH (2016). Electrothermal MEMS fiber scanner for optical endomicroscopy. Opt Express.

[14] Hwang K, Seo Y-H, Jeong K-H (2017). Microscanners for optical endomicroscopic applications. Micro and Nano Systems Letters.

[15] Piyawattanametha W (2009). *In vivo* brain imaging using a portable 2.9 g two-photon microscope based on a microelectromechanical systems scanning mirror. Optics Letters.

[16] Zong, W. J. *et al*. Fast high-resolution miniature two-photon microscopy for brain imaging in freely behaving mice (vol 14, pg 713, 2017). *Nat Methods* **14** (2017). 10.1038/nmeth.4305 28553965

[17] Dunkers JP, Sanders DP, Hunston DL, Everett MJ, Green WH (2002). Comparison of optical coherence tomography, X-ray computed tomography, and confocal microscopy results from an impact damaged epoxy/E-glass composite. J Adhesion.

[18] Denk W, Strickler JH, Webb WW (1990). Two-photon laser scanning fluorescence microscopy. Science.

[19] Berland KM, So PT, Gratton E (1995). Two-photon fluorescence correlation spectroscopy: method and application to the intracellular environment. Biophys J.

[20] Gibson EA, Masihzadeh O, Lei TC, Ammar DA, Kahook MY (2011). Multiphoton microscopy for ophthalmic imaging. J Ophthalmol.

[21] Duan X (2015). MEMS-based multiphoton endomicroscope for repetitive imaging of mouse colon. Biomed Opt Express.

[22] Duan XY, Li HJ, Li X, Oldham KR, Wang TD (2017). Axial beam scanning in multiphoton microscopy with MEMS-based actuator. Opt Express.

[23] Piyawattanametha W (2006). Fast-scanning two-photon fluorescence imaging based on a microelectromechanical systems two- dimensional scanning mirror. Opt Lett.

[24] Fu L, Jain A, Xie H, Cranfield C, Gu M (2006). Nonlinear optical endoscopy based on a double-clad photonic crystal fiber and a MEMS mirror. Opt Express.

[25] Engelbrecht CJ, Johnston RS, Seibel EJ, Helmchen F (2008). Ultra-compact fiber-optic two-photon microscope for functional fluorescence imaging *in vivo*. Opt Express.

[26] Flusberg BA, Lung JC, Cocker ED, Anderson EP, Schnitzer MJ (2005). *In vivo* brain imaging using a portable 3.9 gram two-photon fluorescence microendoscope. Optics Letters.

[27] Do, D., Yoo, H. & Gweon, D. G. Fiber-optic raster scanning two-photon endomicroscope using a tubular piezoelectric actuator. *Journal of Biomedical Optic*s **1**9, 10.1117/1.Jbo.19.6.066010 (2014). 10.1117/1.JBO.19.6.066010 24972358

[28] Sawinski, J. & Denk, W. Miniature random-access fiber scanner for *in vivo* multiphoton imaging. *J Appl Phy*s **102**, 10.1063/1.2763945 (2007).

[29] Bao H, Allen J, Pattie R, Vance R, Gu M (2008). Fast handheld two-photon fluorescence microendoscope with a 475 microm x 475 microm field of view for *in vivo* imaging. Opt Lett.

[30] Wu Y, Leng Y, Xi J, Li X (2009). Scanning all-fiber-optic endomicroscopy system for 3D nonlinear optical imaging of biological tissues. Opt Express.

[31] Ducourthial, G. *et al*. Development of a real-time flexible multiphoton microendoscope for label-free imaging in a live animal. *Sci Rep-Uk* **5**, 10.1038/srep18303 (2015). 10.1038/srep18303 PMC4682136 26673905

[32] Liang W (2012). Increased illumination uniformity and reduced photodamage offered by the Lissajous scanning in fiber-optic two-photon endomicroscopy. J Biomed Opt.

[33] Duan, C. *et al*. An endoscopic forward-viewing OCT imaging probe based on a two-axis scanning mems mirror. *in Biomedical Imaging (ISBI)*, *2014 IEEE 11th International Symposium on*, 1397–1400 (2014).

[34] El Rifai, O. M. & Youcef-Toumi, K. In *American Control Conference*, *2001*. *Proceedings of the 2001*. 3251–3255 (IEEE).

[35] Park HC (2014). Micromachined tethered silicon oscillator for an endomicroscopic Lissajous fiber scanner. Opt Lett.

[36] Hwang, K., Seo, Y. H., Ahn, J., Kim, P. & Jeong, K. H. Frequency selection rule for high definition and high frame rate Lissajous scanning. *Sci Rep-Uk* **7**, 10.1038/s41598-017-13634-3 (2017). 10.1038/s41598-017-13634-3 PMC5658369 29074842

[37] Park HC, Seo YH, Jeong KH (2014). Lissajous fiber scanning for forward viewing optical endomicroscopy using asymmetric stiffness modulation. Opt Express.

[38] Hwang, K. *et al*. Fully packaged confocal endomicroscopic system using Lissajous fiber scanner for indocyanine green *in-vivo* imaging. Proceedings of SPIE BiOS 10497 (2018**)**.

