# Supplementary material for: Lissajous Scanning Two-photon Endomicroscope for In vivo Tissue Imaging
Source: Sci Rep. 2019 Mar 5;9:3560. doi: 10.1038/s41598-019-38762-w (PMC6401070; doi:10.1038/s41598-019-38762-w)
Supplement: Supplementary file 1 — Supplementary information [file 41598_2019_38762_MOESM1_ESM.pdf]

Supplementary information

Lissajous Scanning Two-photon Endomicroscope for *In vivo* Tissue Imaging

Daniel Youngsuk Kim[1,4,+], Kyungmin Hwang[1,4,+], Jinhyo Ahn[2,4], Yeong-Hyeon Seo[1,4], Jae-Beom Kim[1,4], Soyoung Lee[3,5], Jin-Hui Yoon[1,4], Eunji Kong[2,4], Yong Jeong[1,4], Sangyong Jon[3,5], Pilhan Kim[2,4], and Ki-Hun Jeong[1,4,*]

[1]Department of Bio and Brain Engineering, KAIST, Daejeon, 34141, Republic of Korea

[2]Biomedical Science and Engineering Interdisciplinary Program, KAIST, Daejeon, 34141, Republic of Korea

[3]Department of Biological Sciences, KAIST, Daejeon, 34141, Republic of Korea

[4] KAIST Institute of Health science and technology, Daejeon, 34141, Republic of Korea

[5] KAIST Institute for the BioCentury, Daejeon, 34141, Republic of Korea

[*]kjeong@kaist.ac.kr

[+]The authors contributed equally to this work.

1. Scanning speed of Lissajous scanning

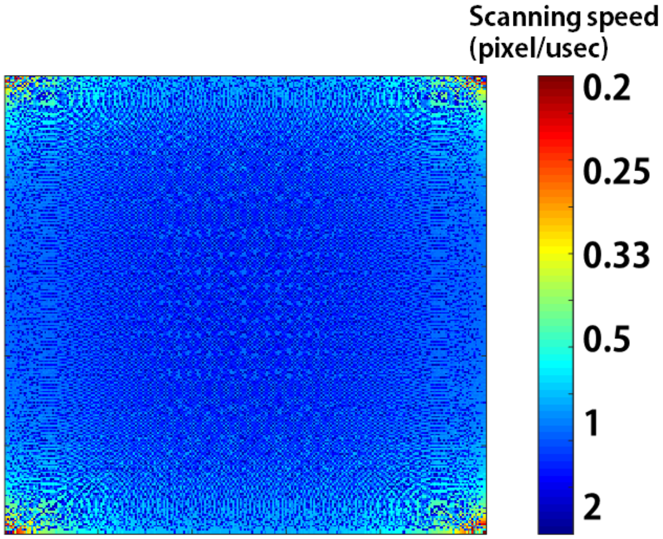

Figure S1. Pixel scanning speed map of 885 Hz and 1160 Hz Lissajous scanning. The map was calculated during 0.2 second at 256 x 256 pixel image. The scanning speed was almost in the range of 0.2 pixel/μs to 2 pixel/μs. The scanning speed is relatively slow at the edge of Lissajous scanning than the other area.

2. Design of group delay dispersion compensable fiber scanner

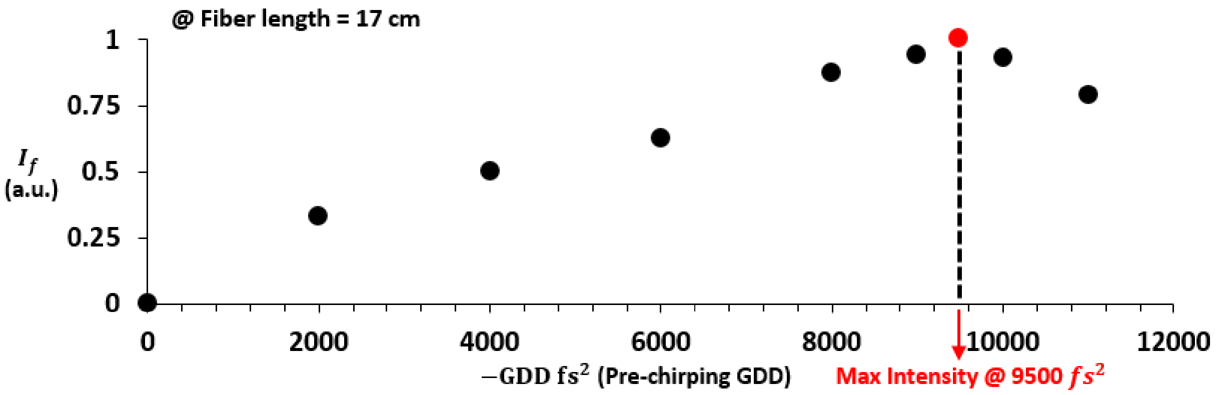

Figure S2 Changes of fluorescence intensity as the negative group delay dispersion (GDD) increased. The positive GDD from the fiber can be compensated by the negative GDD from the prism array. The maximum negative GDD that can be provided by the prism array was 11000 fs2. Since the positive GDD of the fiber core was 3000 fs2/mm and optical components before the fiber was 3000 fs2, the length of the fiber in the scanner was set to 17 cm. As the positive GDD was compensated by the negative GDD, the fluorescence intensity also increased, and when the negative GDD became 9500 fs2, all the positive GDD was compensated and the fluorescence intensity became maximum. After the point, the negative GDD passed the positive GDD, so the pulse width was broaden again and the fluorescence intensity decreased. It shows that the pre-chirp system completely compensated the positive GDD of the 17 cm fiber.

2. Fabrication procedure of the PDMS Pattern

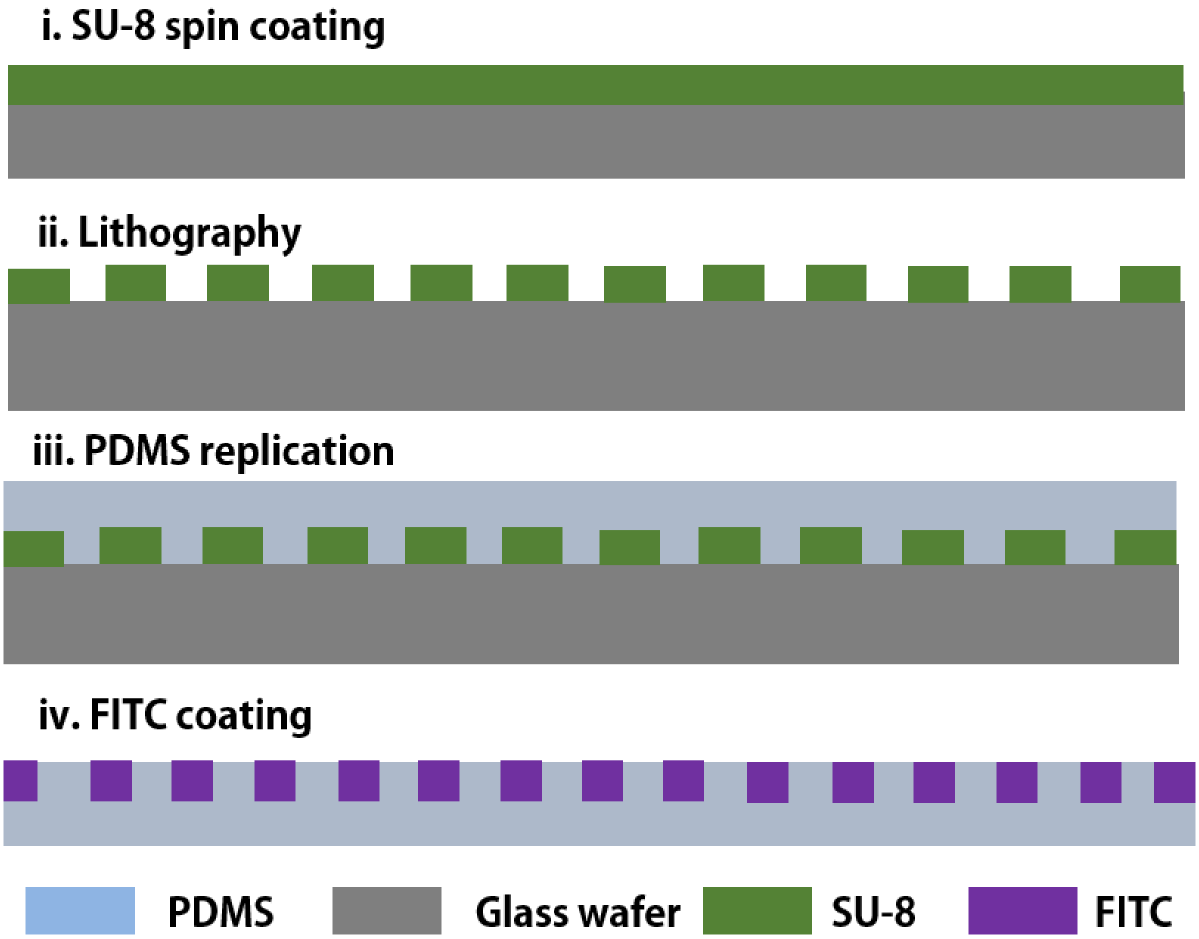

Figure S3. Fabrication procedure of United States Air Force (USAF) test target patterned PDMS coated with fluorescein isothiocyanate (FITC). SU-8 photoresist was coated on a glass wafer, and photolithography was proceeded with the USAF resolving power test target 1951 patterned mask. The USAF test target pattern was replicated on the PDMS with FITC coating.

3. Simulation of beam expansion for coupling into the core of the fiber

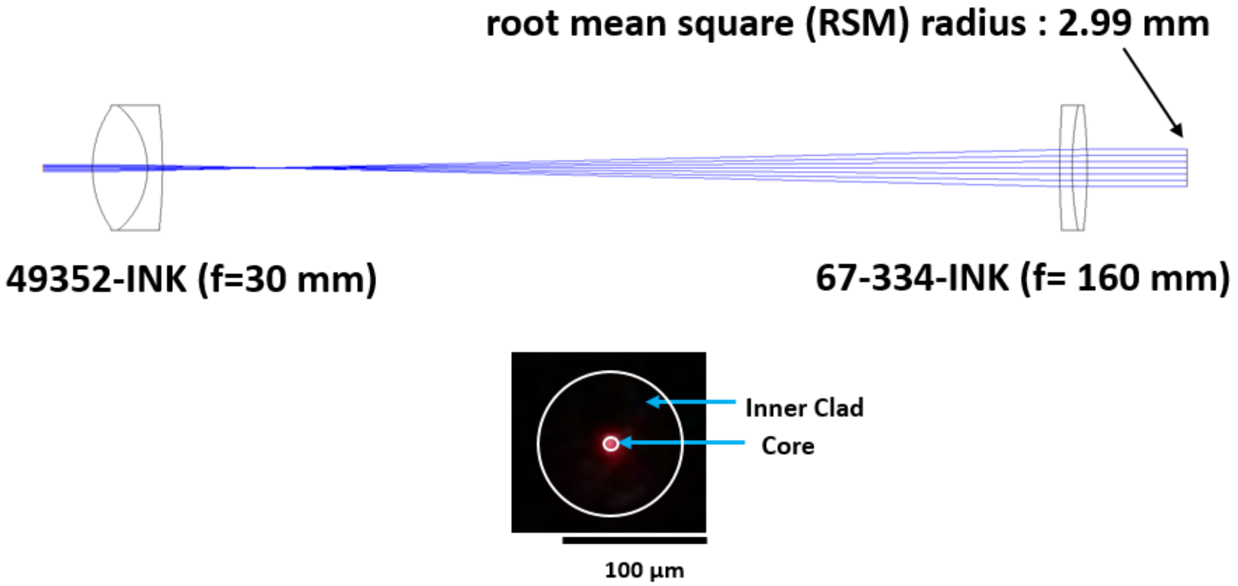

Figure S4. Expanding excitation laser for coupling into the core of the fiber. The laser was expanded to 2.99 mm in root mean square (RSM) radius (Zemax, LLC) by using two achromatic lenses (f=30 mm, 49352-INK, Edmund Optics), (f= 160 mm, 67-334-INK, Edmund Optics). The expanded beam was focused by using a microscope objective lens (RMS X10, Thorlabs) and only coupled into the core of the fiber.
